# Association between climate variables and pulmonary tuberculosis incidence in Brunei Darussalam

**DOI:** 10.1038/s41598-022-12796-z

**Published:** 2022-05-24

**Authors:** Liling Chaw, Sabrina Q. R. Liew, Justin Wong

**Affiliations:** 1 PAPRSB Institute of Health Sciences, Universiti Brunei Darussalam, Bandar Seri Begawan, Brunei Darussalam; 2 College of Human Ecology, Cornell University, Ithaca, USA; 3 Disease Control Division, Ministry of Health, Bandar Seri Begawan, Brunei Darussalam

**Keywords:** Environmental sciences, Diseases

## Abstract

We investigated the association between climate variables and pulmonary tuberculosis (PTB) incidence in Brunei-Muara district, Brunei Darussalam. Weekly PTB case counts and climate variables from January 2001 to December 2018 were analysed using distributed lag non-linear model framework. After adjusting for long-term trend and seasonality, we observed positive but delayed relationship between PTB incidence and minimum temperature, with significant adjusted relative risk (adj.RR) at 25.1 °C (95th percentile) when compared to the median, from lag 30 onwards (adj.RR = 1.17 [95% Confidence Interval (95% CI): 1.01, 1.36]), suggesting effect of minimum temperature on PTB incidence after 30 weeks. Similar results were observed from a sub-analysis on smear-positive PTB case counts from lag 29 onwards (adj.RR = 1.21 [95% CI: 1.01, 1.45]), along with positive and delayed association with total rainfall at 160.7 mm (95th percentile) when compared to the median, from lag 42 onwards (adj.RR = 1.23 [95% CI: 1.01, 1.49]). Our findings reveal evidence of delayed effects of climate on PTB incidence in Brunei, but with varying degrees of magnitude, direction and timing. Though explainable by environmental and social factors, further studies on the relative contribution of recent (through primary human-to-human transmission) and remote (through reactivation of latent TB) TB infection in equatorial settings is warranted.

## Introduction

The seasonal variation of tuberculosis (TB) is long known but less understood phenomenon^1^. Many ecological studies have investigated the relationship between TB incidence and climate variables (relating to temperature, humidity, rainfall, sunlight and wind speed) in temperate^2,3^, subtropical^4,5^,and tropical^6,7^ countries. These studies investigated whether climate factors have any effect on the predominantly airborne transmission of *Mycobacterium tuberculosis* (MTB), the causative agent of TB. Experiments suggested that the quantity of small droplets or aerosolized particles containing MTB produced by a TB patient is predictive of infection among household contacts^8^. Also, conditions of high relative humidity could facilitate small droplets containing MTB to evaporate slowly, thus allowing it to remain suspended in the air for a longer period of time^9^. It was previously reported that ultra-violet light can prevent TB transmission^10^, and that relative humidity and wind speed^11^ can impact the risk of airborne transmission.

However, findings of these ecological studies tend to be contradictory. This could be explained by the substantial and known heterogeneity in disease distribution, due to factors such as the geographic variation of disease burden, individual-level factors for the infectious and susceptible host, potential time delays in healthcare-seeking and diagnosis, and social mixing patterns in a particular setting^12^. This is further complicated by the fact that successful transmission of MTB could result in different TB disease manifestations, leading to different timings for symptoms to appear. The median incubation period of TB was found to be 1.3 years^13^, where most secondary contacts develop active TB disease within several months to 2 years^14^.

Time-series analyses^2,3,15^, as well as techniques incorporating spatial data and regression^7,16^, have been used to study the relationship between climate variables and TB diagnosis. Due to the relatively long incubation period for TB, it is also important to take in account the delayed and non-linear effects of climate variables when studying TB seasonality. This can be done using the distributed lag non-linear model (DLNM) framework, which was previously applied to TB^4,5,17^ and other diseases such as acute respiratory infections^18^, Zika^19^, and hand, foot and mouth disease^20^.

Brunei Darussalam (pop. 453,600^21^) is a small Southeast Asian country that is located at about 4°N north of the Equator. The country has a tropical equatorial climate where it is generally hot and wet throughout the year, and with little seasonal variation. Most of the population live close to the coastal plain regions. The country has also has an intermediate TB burden, with an incidence of 57 per 100,000 population in 2017^22^. While association studies between TB and climate variables from equatorial regions are few, conflicting reports were still observed^1^, ranging from little seasonal variability in neighboring Singapore (4°N) to some seasonal variation from studies in Cameroon (4°N)^23^ and northern Lima, Peru (12°S)^24^.

This study aims to investigate any association between climate variables and PTB incidence in Brunei. We decided to focus on PTB due to its infectiousness and that it can be caused by human-to-human transmission. Our findings could also be helpful to identify any patterns to inform future public health interventions and contribute to the current knowledge on the topic in locations near to the Equator.

## Materials and methods

### Data collection

Weekly case counts of all diagnosed PTB cases who resided in the Brunei-Muara district, Brunei between January 2001 and December 2018 (18 years, 939 weeks) were compiled from the National TB Coordinating Centre (NTCC). Brunei-Muara district is the most populated district in the country where 69.7% of the population reside^21^, and where the capital city is located. NTCC was established as part of the National TB programme in Brunei, and has implemented TB surveillance, treatment and control programmes since 2000. All patients suspected to have any form of TB across the whole country are often referred to NTCC, or any respective district directly observed treatment, short course (DOTS) centre, for diagnosis, treatment and follow-up^25^. All modes of diagnosis for the PTB cases were included (such as smear-positive, smear-negative, and through chest X-ray and/or clinician’s decision). These case counts were summed up by epidemiological week and year, based on treatment start date. In cases where the treatment start date is missing, the NTCC registration date was used.

Daily data on climate variables for the same period were obtained from a local meteorological station, located at Brunei-Muara district. The variables provided includes total sunshine hours, total rainfall (in millimeters), average wind speed (in knots), relative humidity (RH) in percentage (minimum, mean and maximum), and temperature in degree Celsius (minimum, mean and maximum). These daily data were averaged by epidemiological week and year. Any missing daily values (n = 5) were replaced with the mean value for that particular month and year. Vapour pressure (a measure of absolute humidity) was calculated using the Clausius-Clapeyron equation^26^, by inputting the mean RH values and the standard temperature and pressure conditions.

### Statistical analysis

Spearman’s rank correlation test was used to explore the correlation between each climate variable, and with PTB case counts. Stationarity of the time series for weekly PTB case counts and each climate variable were checked using the augmented Dickey-Fuller test.

We used distributed lag non-linear model (DLNM) framework to investigate the association between climate variables and PTB incidence. Under this model framework, negative binomial distribution was assumed to account for overdispersion, and crossbasis terms were constructed for each climate variables. These terms comprise of 2 dimensions: one specifying the conventional exposure–response relationship, and the other specifying the lag-response relationship^27^. Natural cubic splines with 7 degrees of freedom (df) per calendar year were used to account for long-term trends and seasonality. This adjustment was included based on previous similar studies for TB^5,17^, and the number of df was determined using the Akaike’s Information Criterion (AIC) value. Natural cubic splines with 3 df were used to describe both the lagged and non-linear effects of each climate variable.

The median incubation period for PTB ranges between few months to 2 years^14^, and there is often a delay in diagnosing TB, by about 5–6 months^25,28^. Considering these factors, we decided to specify lags of up to 12 months (52 weeks) to capture the delayed effects of climate variables. The rationale is to cover as much of the incubation period without sacrificing any loss of statistical accuracy and efficiency that could be caused by adding more lags^4,17^. The general model formula structure used is as follows:

$$log E\left( {Y_{t} } \right) = \alpha + \sum CB \left( {M,lag} \right) + ns\left( {t,df = 7/year \times no. of years} \right)$$

where E(Y_t_) is the expected number of PTB cases at week t, $\alpha$ is the intercept, CB is the cross-basis function used for each climate variable to be assessed (M**)**, and ns is the natural cubic spline function applied to account for long-term trend and seasonality. The presence of any residual auto-correlation were assessed using partial autocorrelation function plots (PACF). Any remaining autocorrelation detected was accounted for by adding lags of the model’s deviance residuals into the final model.

Although not all variables give significant results during univariate analysis, we decided to include 5 crossbasis terms that represent different aspects of climate variables and that are also previously known to be associated with TB incidence. The rationale here is to include these variables to control for potential confounding. The AIC value was used to assess which variables to be included in final model. This resulted in the choice of the following 5 variables in the final model: average wind speed, total sunshine hours, total rainfall, mean RH and minimum temperature. To ensure minimal issues with multi-collinearity and/or correlation (due to the use of multiple crossbasis terms in a single model), consistency in results obtained between univariate and multivariate were checked using visual analysis and referring to the AIC value.

We reported the relative risk (RR) of weekly PTB cases at the 5th and 95th percentiles of each climate variable, compared to their median, with corresponding 95% confidence intervals (95% CI). For climate variables with significant results observed at either percentile, we further determined and reported the starting lag week at which this significant result can be found. Overall relationship patterns were also described using three-dimensional (3D) and contour plots. Lag plots were used to show trend differences at lags 0, 13, 26, 39 and 52 weeks (corresponding to 0, 3, 6, 9 and 12 months), with higher number of lags indicating longer lagged effect of the corresponding climate variable As an additional sub-analysis, we repeated the same analysis described above to report the RR of weekly smear-positive PTB cases. Sensitivity analyses were conducted by repeating the analysis using natural cubic splines of 5 and 9 df for long-term trend. All analyses were done and all figures were generated in R (ver. 4.1), using tseries, splines and dlnm packages^29,30^.

This study was approved by the Medical and Health Research and Ethics Committee (MHREC), Ministry of Health, Brunei (Ref: MHREC/UBD/2019/2). All methods were performed in accordance with the relevant guidelines and regulations. Informed consent was waived because all analyses were based on aggregated data which do not contain any identifying or sensitive information.

## Results

**Figure 1:**
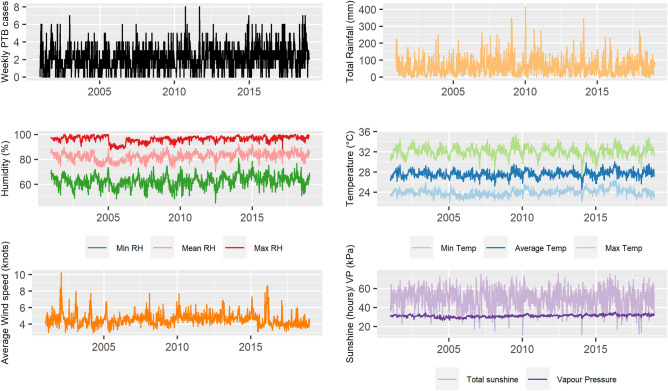
Time series for weekly PTB case counts and climate variables from January 2001 to December 2018 at Brunei-Muara district, Brunei.

A total of 1967 PTB cases were reported from January 2001 to December 2018, out of which 1412 (71.8%) were smear-positive PTB cases. The median number of weekly PTB cases was 2 (IQR = 1–3), ranging between 0 and 8. Table 1 and Fig. 1 shows the summary statistics and time series of each climate variable and weekly PTB case counts.

**Table 1:** Summary values for all climate variables and PTB case counts, Brunei (2001–2018).

Variables	Minimum	5th percentile	Mean	sd	Median	IQR	95th percentile	Maximum
Average wind speed (knots)	3.0	3.6	4.6	0.81	4.5	4.0–4.9	6.1	10.2
Total sunshine (hours)	11.2	30.8	49.6	11.05	50.7	42.4–57.5	66.1	76.0
Total rainfall (mm)	0.0	0.8	61.1	56.15	44.9	18.4–91.8	160.7	414.5
Minimum RH (%)	45.0	53.7	62.6	4.96	63.0	59.4–65.8	70.3	80.6
Mean RH (%)	70.0	76	82.5	3.43	82.7	80.3–84.9	87.6	92.1
Maximum RH (%)	87.7	91	96.2	2.45	96.9	95.1–98.0	99.3	100.0
Vapour pressure (kPa)	26.4	28.8	31.1	1.31	31.2	30.4–32.0	33.2	35.1
Minimum temperature (°C)	21.8	22.9	24.0	0.68	24.0	23.5–24.4	25.1	26.3
Average temperature (°C)	24.5	26.5	27.6	0.72	27.6	27.2–28.1	28.9	30.0
Maximum temperature (°C)	27.1	30.6	32.1	1.00	32.1	31.5–32.7	33.7	35.5
Weekly PTB case counts	0.0	0	2.1	1.52	2.0	1–3	5	8.0
Weekly smear-positive PTB case counts	0	0	1.5	1.26	1	1–2	4	8

Spearman’s rank correlation test (S1 Table) shows a significant but weakly positive association between PTB and humidity-related variables (specifically mean RH, maximum RH and vapour pressure). Moderate and significant association were generally observed between climate variables, with stronger association seen among related variables (such as for temperature and humidity).

**Figure 2:**
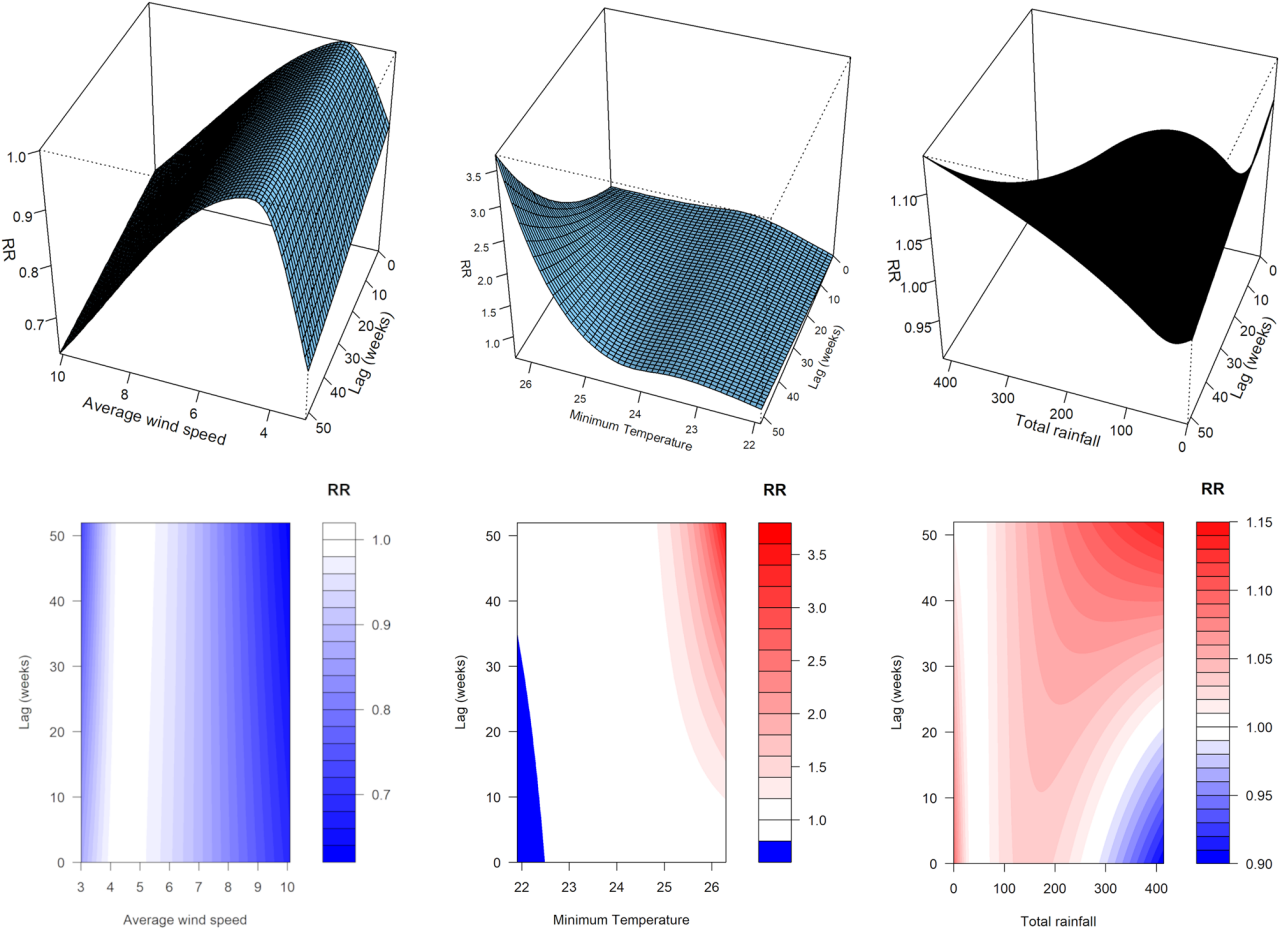
Three-dimensional exposure-lag-response curves (top) and corresponding contour plots (bottom) from final model, for (left to right) average wind speed, minimum temperature and total rainfall.

**Figure 3:**
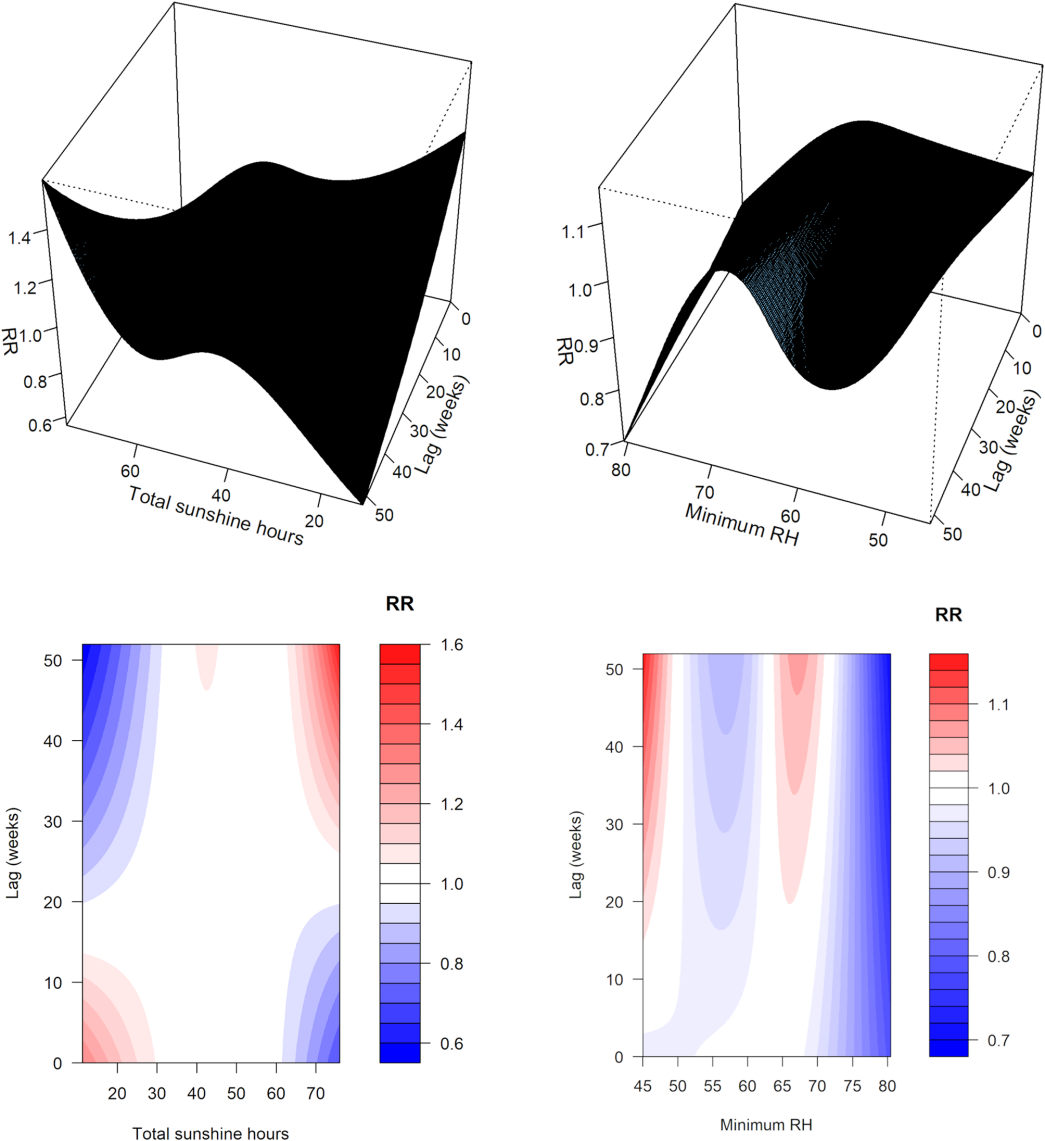
Three-dimensional exposure-lag-response curves (top) and corresponding contour plots (bottom) from final model, for total sunshine hours (left) and minimum relative humidity (right).

Figures 2 and 3 show the overall exposure-lag-response relationship of PTB and climate variables included in the final model, using 3D and contour plots. It is apparent that different variables affect weekly PTB incidence differently, with the shape of the interaction changing with increasing lags.

**Figure 4:**
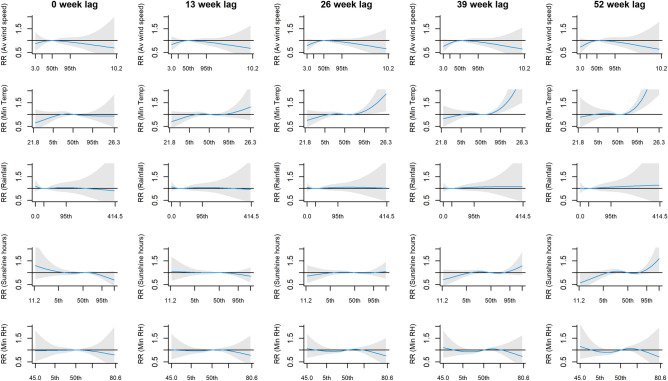
The lagged effects on the relative risk (RR) of PTB incidence from final multivariate model, of (from top to bottom) average wind speed, minimum temperature, total rainfall, total sunshine hours and minimum relative humidity, at lags 0, 13, 26, 39 and 52 weeks. There are 5 x-axis label ticks for each plot, corresponding to the minimum, 5th percentile, 50th percentile, 95th percentile and maximum. The areas shaded in grey correspond to the 95% Confidence Interval.

After adjusting for long term trend and seasonality, we observed positive but delayed relationship between PTB incidence and minimum temperature, with adjusted RR (adj. RR) at 25.1 °C (95th percentile) when compared to the median from lag 30 onwards (adj. RR = 1.17 [95% Confidence Interval (95% CI): 1.01, 1.36], adj. RR = 1.25 [95% CI: 1.07, 1.48] and adj. RR = 1.38 [95% CI: 1.13, 1.70] at lags 30, 39 and 52, respectively; Fig. 4). Although no significant findings were observed for the other variables, we observed a flat and inverted U-shaped across lags for average wind speed, a relatively flat relationship across lags for total rainfall, and the emergence of inverted S-shaped pattern for total sunshine hours and minimum RH towards the later lags.

**Figure 5:**
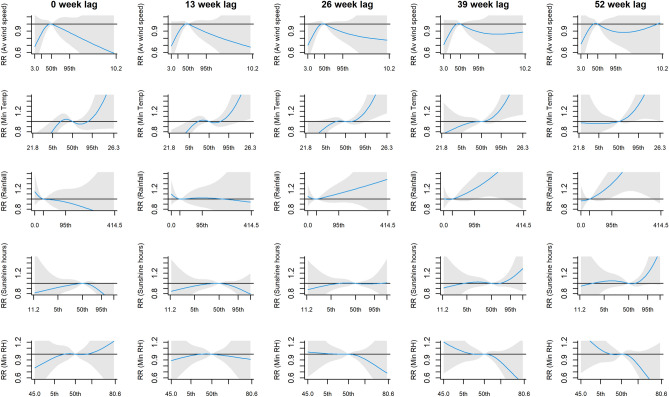
The lagged effects on the relative risk (RR) of smear-positive PTB incidence from final multivariate model, of (from top to bottom) average wind speed, minimum temperature, total rainfall, total sunshine hours and minimum relative humidity, at lags 0, 13, 26, 39 and 52 weeks. There are 5 x-axis label ticks for each plot, corresponding to the minimum, 5th percentile, 50th percentile, 95th percentile and maximum. The areas shaded in grey correspond to the 95% Confidence Interval.

Similar result for minimum temperature was found when the analysis was repeated using weekly counts of smear-positive PTB cases, where the significant adjusted RR was observed at the 25.1 °C (95th percentile) from lag 29 onwards (adj. RR = 1.21 [95% CI: 1.01, 1.45], adj. RR = 1.26 [95% CI: 1.03, 1.54] and adj. RR = 1.33 [95% CI: 1.04, 1.70] at lags 29, 39 and 52, respectively). In addition, total rainfall was found to be associated with smear-positive PTB; adjusted RR was also significantly high at the 160.7 mm rainfall (95th percentile) from lag 42 onwards (adj RR = 1.23 [95% CI: 1.01, 1.49] and adj RR = 1.30 [95% CI: 1.03, 1.65] at lags 42 and 52, respectively; Fig. 5).

Univariate results for each climate variable follow a similar trend and direction of association when compared to the final multivariable model (S1 Fig), indicating minimal multicollinearity issues. Although statistical significance for minimum temperature was lost when sensitivity analysis was done using natural cubic splines (ns) of 5 and 9 df to account for long-term trend, the trend and direction of association remained similar (S2 Fig and S3 Fig).

## Discussion

In this study, we used a multivariable negative binomial model with DLNM framework to investigate the association between climate variables and PTB incidence in Brunei. We found a positive but delayed relationship with minimum temperature. Also, as the number of lagged weeks increased, we observed varying degrees of magnitude, direction and timing across all evaluated climate variables.

Our finding for minimum temperature was consistent with similar studies from Vietnam^7^ and Hong Kong^5^. This result remained consistent for the sub-analysis on smear-positive PTB case counts, where we also observed an additional positive but delayed relationship with total rainfall. A plausible explanation for this finding relates to changes in human activities in both indoor and outdoor settings in response to temperature changes and rainfall, which in turn could lead to differences in the human-to-human transmission of MTB^4^. Such effects can be notably observed in Ethiopia, where the rainy season negatively affects healthcare-seeking behaviour for chronic illnesses^6^. It should be noted, however, that our findings tend to be significant only at extreme values for the specific climate variable (in our case, at the 95% percentile). This suggests either a very minimal effect of climate variables on PTB incidence under “normal” climate conditions, or that it is difficult to tease out the effect of climate variable in locations with minimal variation in climate variables; both explanations which could be applicable to the equatorial Brunei setting.

Although not statistically significant, we observed the formation of an inverted S-shaped trend for total sunshine as the number of lags increases, suggesting a probable inverse relationship between PTB incidence and low sunshine hours after a lag of 9 months or higher. This finding contrasts to those found in countries with particularly temperate and subtropical climates, where TB incidence peak in spring/summer, and trough in autumn/winter^1,7^. Other studies suggest a relationship between vitamin D deficiency in winter months, a proxy for the lack of sunshine hours, with high TB notifications 3–6 months later^31,32^. Our finding could be due to the fact that Brunei lies near to the equator, where variations in daily sunlight exposure throughout the year are minimal. Indeed, multiple studies have shown stronger seasonality patterns in areas further from the Equator^1^. Further, although a Vietnamese study^7^ reported similar results to that in temperate and subtropical countries, the authors also noted that individuals in Vietnam often take measures to prevent sun exposure; a point that was not considered in their analysis, and is also a relevant limitation to the Bruneian setting. Hence, a plausible explanation for our finding could be again related to changes in human activities at both indoor and outdoor settings.

Understanding PTB seasonality is complex due to the ways it could manifest, that is either recently through exogenous infection, or remotely through endogenous reactivation of latent TB infection. It is still undetermined whether variation in climate factors affects either or both types of infection, though recent infection is likely^1^. While TB cases in high and low TB burden areas tend to be driven, respectively, by recent and remote infection^14,33^, it is unclear where areas with intermediate TB burden, such as Brunei, stands. While our study findings and their possible explanations point more towards climate factors affecting human-to-human transmission, and thus leading to recent infections, it is still premature to rule out its contribution to remote infection. Future simulation studies on the driving factor in Brunei and/or equatorial countries could help to shed light on this. Also, it should be noted that our study findings could be more applicable to outdoor humidity conditions. Due to the daily hot and humid conditions in Brunei, the use of indoor air-conditioning is very common albeit for households who can afford it. Further studies that could incorporate data on air-conditioning usage in households or collecting socio-economic status data, or focusing on indoor climate conditions instead would help to determine the role played by RH and temperature in the human-to-human transmission of MTB.

Our study has several limitations. Firstly, our findings cannot be translated at the individual level as this is an ecological study. Secondly, we analysed data only from Brunei-Muara district because complete climate data was only available locally from a single meteorological station located in that district. Thirdly, we did not incorporate non-climatic factors that are also known risk factors of PTB, such as age, presence of co-morbidities and socio-economic status, which could confound the study results. The main strength of our study is the availability of an 18-year long dataset with weekly intervals. Analysing using time series with shorter time intervals could possibly lead to more accurate results. Also, we opted to report the multivariate model results for 5 crossbasis terms encompassing the different aspects of climate, which would theoretically control for possible confounding.

In conclusion, we found a positive but delayed relationship between PTB incidence and minimum temperature. As the number of lagged weeks increased, we observed varying degrees of magnitude, direction and timing across all evaluated climate variables. These variations could be explained by environmental and social factors, mainly affecting human-to-human transmission. To better understand this relationship, future studies on the relative contribution of recent and remote TB infection in equatorial settings is warranted.

## Supplementary Information

Supplementary Information 1.

Supplementary Information 2.

## Data Availability

All data generated or analysed during this study are included in this published article [and its supplementary information files]. The codes used during the analysis are available in the Rubs platform [https://rpubs.com/LilingC/889472].
