# Supplementary material for: Association between climate variables and pulmonary tuberculosis incidence in Brunei Darussalam
Source: Sci Rep. 2022 May 24;12:8775. doi: 10.1038/s41598-022-12796-z (PMC9130123; doi:10.1038/s41598-022-12796-z)

**Supplementary Information**

**For manuscript title:** Association between climate variables and pulmonary tuberculosis incidence in Brunei Darussalam, 2001 – 2018

Liling Chaw^1^, Sabrina Q.R. Liew^2^, Justin Wong^3^

S1 File. Dataset used for this study.

S1 Table. Spearman’s rank correlation test results.

| Variables | Average wind speed | Total sunshine | Total rainfall | Minimum RH | Mean RH | Maximum RH | Vapour pressure | Minimum temp | Average temp | Maximum temp |
| --- | --- | --- | --- | --- | --- | --- | --- | --- | --- | --- |
| Average wind speed | 1 |  |  |  |  |  |  |  |  |  |
| Total sunshine | -0.01 | 1 |  |  |  |  |  |  |  |  |
| Total rainfall | 0.05 | **-0.26** | 1 |  |  |  |  |  |  |  |
| Minimum RH | **0.13** | **-0.3** | **0.38** | 1 |  |  |  |  |  |  |
| Mean RH | 0.06 | **-0.33** | **0.49** | **0.79** | 1 |  |  |  |  |  |
| Maximum RH | **-0.16** | **-0.13** | **0.46** | **0.53** | **0.68** | 1 |  |  |  |  |
| Vapour pressure | **-0.13** | **0.25** | **0.04** | **0.23** | **0.43** | **0.34** | 1 |  |  |  |
| Minimum temp | 0.02 | **0.34** | **-0.35** | -0.04 | **-0.08** | **-0.1** | **0.65** | 1 |  |  |
| Average temp | **-0.17** | **0.57** | **-0.42** | **-0.52** | **-0.49** | **-0.32** | **0.51** | **0.7** | 1 |  |
| Maximum temp | **-0.22** | **0.45** | **-0.32** | **-0.77** | **-0.59** | **-0.35** | **0.25** | **0.39** | **0.82** | 1 |
| PTB case counts | -0.04 | -0.01 | 0.01 | 0.03 | **0.08** | 0.05 | **0.11** | 0.04 | 0.02 | 0 |
| Smear-positive case counts | -0.02 | 0.01 | -0.02 | -0.03 | -0.02 | -0.02 | 0.01 | 0.04 | 0.03 | 0.03 |

RH = Relative Humidity; Bold values indicates that p < 0.05

S1 Fig. The lagged effects on the relative risk (RR) of PTB incidence from univariate model, of (from top to bottom) average wind speed, minimum temperature, total rainfall, total sunshine hours and minimum relative humidity, at lags 0, 13, 26, 39 and 52 weeks. There are 5 x-axis label ticks for each plot, corresponding to the minimum, 5^th^ percentile, 50^th^ percentile, 95^th^ percentile and maximum.

**
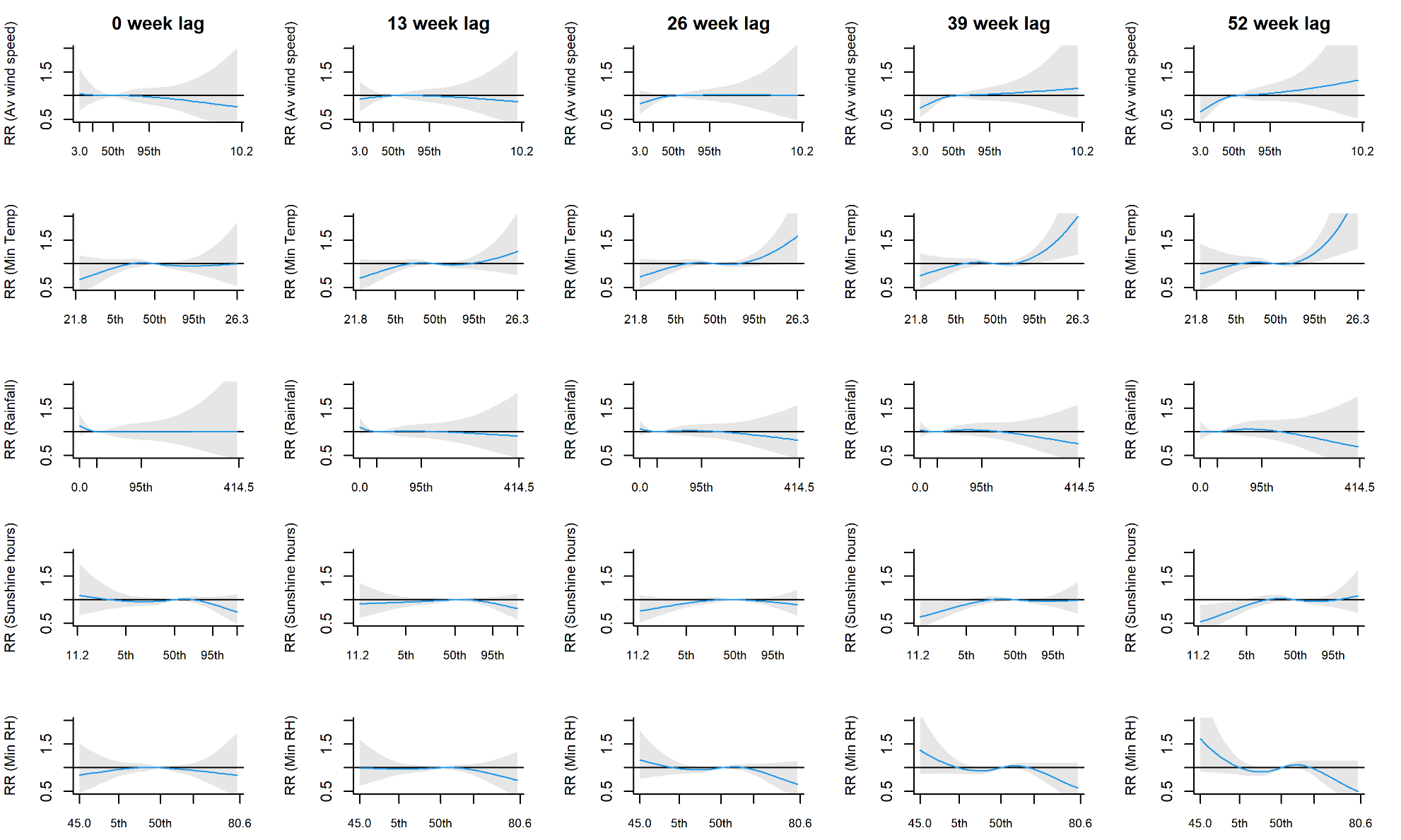
**

S2 Fig. Sensitivity analysis with natural cubic spline of 5df for long-term trend: The lagged effects on the relative risk (RR) of PTB incidence from final multivariate model, of (from top to bottom) average wind speed, minimum temperature, total rainfall, total sunshine hours and minimum relative humidity, at lags 0, 13, 26, 39 and 52 weeks. There are 5 x-axis label ticks for each plot, corresponding to the minimum, 5^th^ percentile, 50^th^ percentile, 95^th^ percentile and maximum.


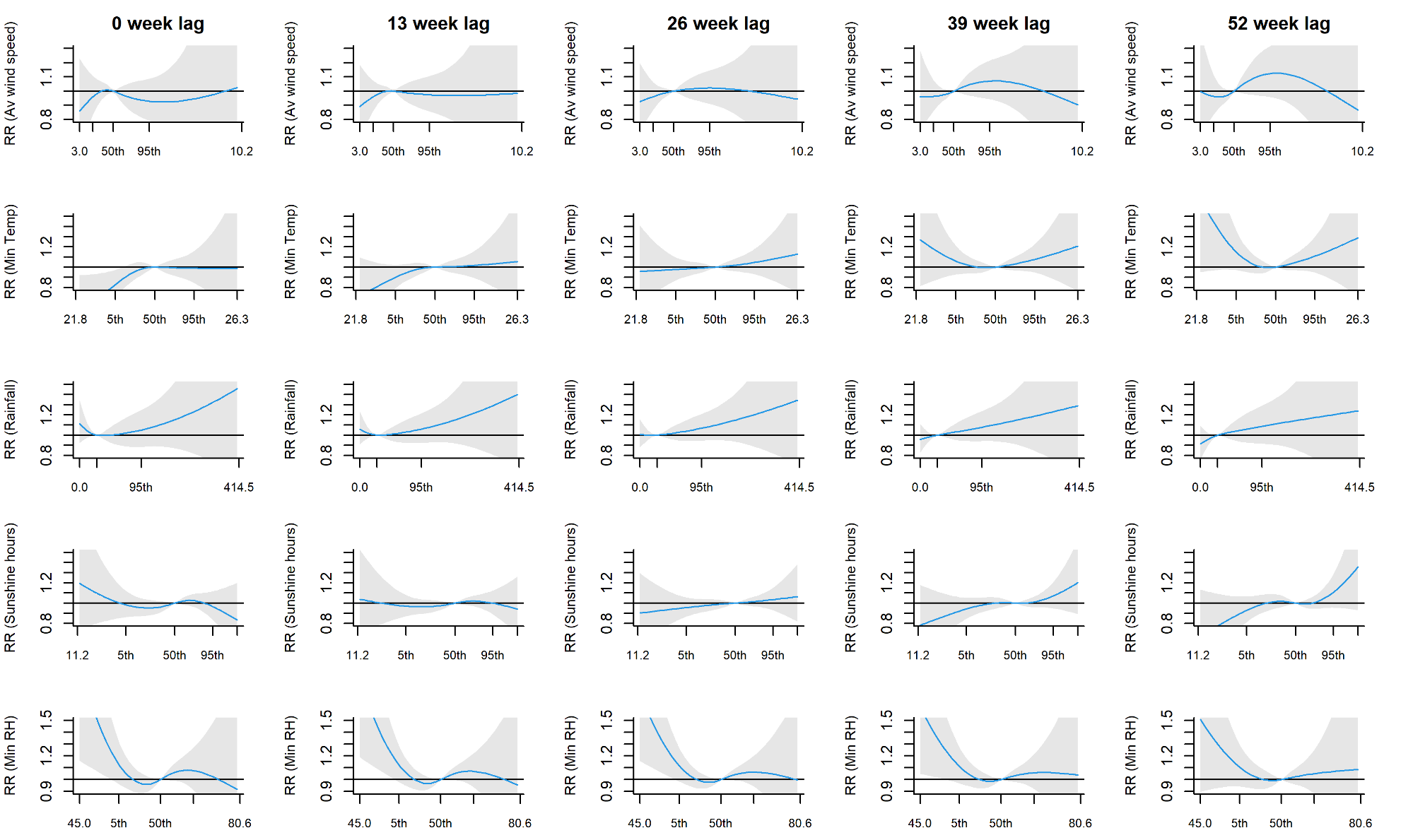


S3 Fig. Sensitivity analysis with natural cubic spline of 9df for long-term trend: The lagged effects on the relative risk (RR) of PTB incidence from final multivariate model, of (from top to bottom) average wind speed, minimum temperature, total rainfall, total sunshine hours and minimum relative humidity, at lags 0, 13, 26, 39 and 52 weeks. There are 5 x-axis label ticks for each plot, corresponding to the minimum, 5^th^ percentile, 50^th^ percentile, 95^th^ percentile and maximum.


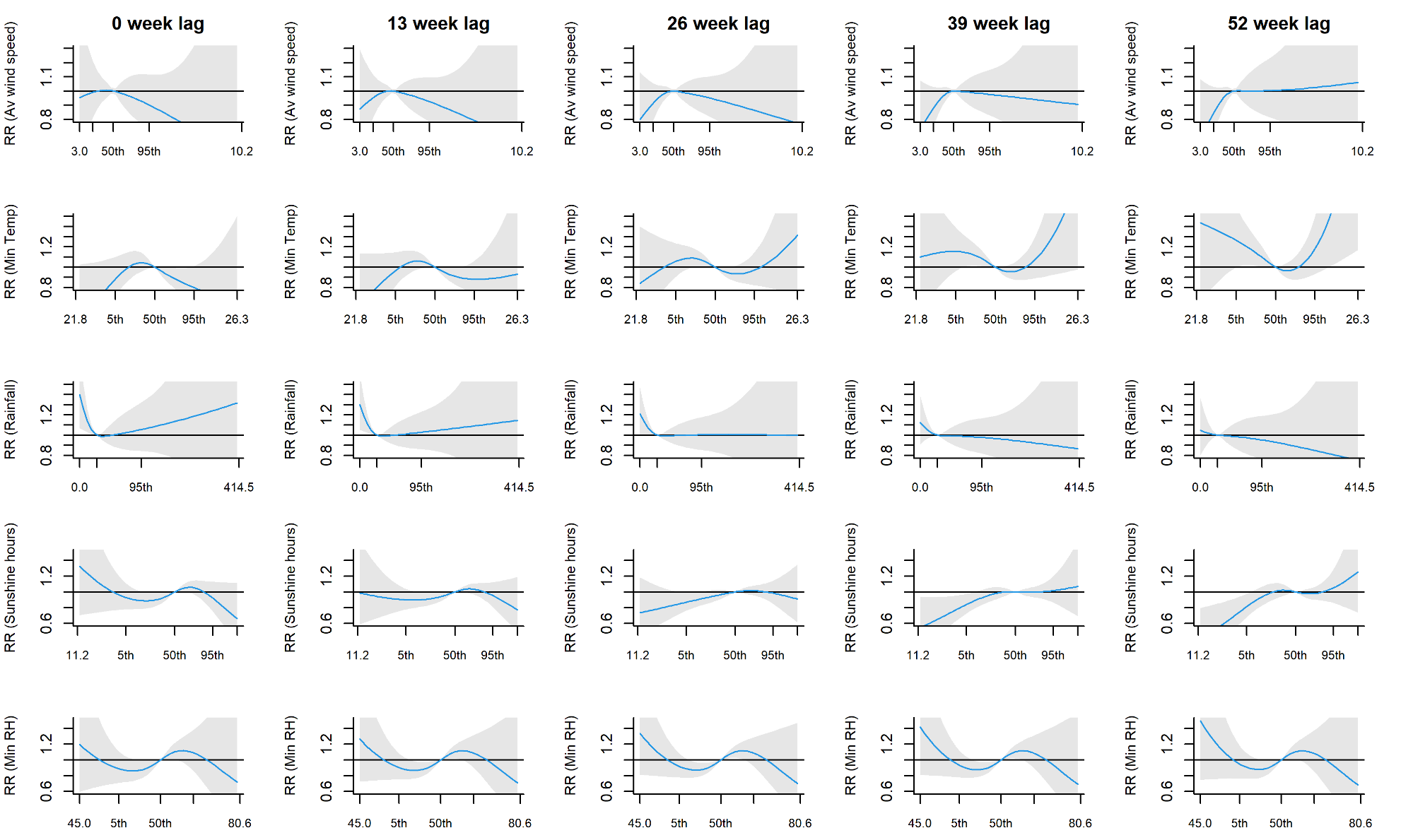

Supplement: Supplementary file 2 — Supplementary Information 2. [file 41598_2022_12796_MOESM2_ESM.docx]
